# ADAM10 and ADAM17 as Biomarkers Linked to Inflammation, Metabolic Disorders and Colorectal Cancer

**DOI:** 10.3390/cimb44100309

**Published:** 2022-09-29

**Authors:** Magdalena Sikora-Skrabaka, Katarzyna Weronika Walkiewicz, Ewa Nowakowska-Zajdel, Dariusz Waniczek, Joanna Katarzyna Strzelczyk

**Affiliations:** 1 Department of Nutrition Related Prevention, Department of Prevention of Metabolic Diseases, Faculty of Health Sciences in Bytom, Medical University of Silesia in Katowice, 40-055 Katowice, Poland; 2 Department of Clinical Oncology, No. 4 Provincial Specialist Hospital, 41-902 Bytom, Poland; 3 Department of Oncological Surgery, Faculty of Medical Sciences in Zabrze, Medical University of Silesia in Katowice, 40-055 Katowice, Poland; 4 Department of Medical and Molecular Biology, Faculty of Medical Sciences in Zabrze, Medical University of Silesia in Katowice, 40-055 Katowice, Poland

**Keywords:** ADAM10, ADAM17, colorectal cancer, biomarkers, diabetes mellitus type 2, cardiovascular diseases, inflammation, metabolic disorders

## Abstract

ADAM10 and ADAM17 have a role in inflammation and diseases associated with inflammation, such as diabetes, cardiovascular diseases (CVD) or cancer, e.g., colorectal cancer (CRC). The aim of this study was to evaluate whether ADAM10 and ADAM17 could be biomarkers of CRC. To achieve this goal, CRC tumors and a surgical margin from 72 patients with CRC were collected. The concentration of ADAM proteins was measured by the ELISA method. Results were analyzed statistically and compared with selected clinical parameters. We found that ADAM17 protein concentration in the tumor samples was higher in patients with diabetes mellitus type 2 (DMT2) (0.28 vs. 0.2 ng/µg protein; *p* = 0.01) and in the surgical margin was higher both in patients with coexisting DMT2 (0.22 vs. 0.16 ng/µg protein; *p* < 0.05) and CVD (0.21 vs. 0.13 ng/µg protein; *p* < 0.01). The concentration of ADAM10 was higher in the surgical margin than in the tumor (249.34 vs. 228.82 pg/µg protein), and the concentration of ADAM17 was higher in the tumor than in the margin (0.23 vs. 0.18 ng/µg protein), but results were not statistically significant. In conclusion, the results of our study indicate that ADAM10 and ADAM17 may be potential biomarkers in cancer linked with DMT2 and CVD as diseases associated with inflammation.

## 1. Introduction

According to the epidemiological data from 2020, colorectal cancer (CRC) ranks third in terms of new cancer cases (after breast cancer and lung cancer) and is the second cause of death among these diseases (after lung cancer) [1]. Confirmed risk factors for the development of CRC are obesity, diet or cigarette smoking and among the mechanisms, inter alia, the activation of chronic inflammatory processes in the intestinal mucosa. Inflammatory Bowel Diseases (IBD) increase the risk of developing a CRC known as colitis-associated cancer (CAC) [2]. It is estimated that the risk of developing CRC in patients with ulcerative colitis after 30 years of disease is 18% [3], and in patients with Crohn’s disease—8.3% [4]. The signaling pathways involved in the pathomechanism of cancer include the pathways of activation of IGFs [5], VEGF, FGF, EGF and those directly related to the activation of chronic inflammatory processes, through the influence of, e.g., TNFα [6,7,8]. These pathways are dependent on the proteins of the ADAM family. The relationship of ADAM proteins with inflammation and carcinogenesis is evidenced, for example, by the results of studies in which obesity has been shown to increase the serum levels of ADAM17 and 28, as well as some pro-inflammatory cytokines such as TNFα (which together lead to the induction of inflammation and tumorigenesis) [9,10]. The relationship between TNFα and ADAM17 levels and obesity is important because ADAM17 is responsible for the biological activity of TNFα. Moreover, activation of ADAM17 in adipose tissue has been shown to lead to the activation of inflammatory molecules such as IL-6 and monocyte chemostatic protein 1 (MCP-1) [10]. One of the pathways involved in the carcinogenesis of colorectal cancer is the signaling pathway related to the activation of EGFR, for which the ligand is, inter alia, transforming growth factor-alpha (TGFα), which is formed with the participation of ADAM 10 and 17 proteins. The tetraspanin-29/ADAM10/Notch pathway also plays an important role in colorectal cancer. Proteins from the tetraspanin family regulate the transport and activity of ADAM10, and ADAM10 acts upstream of Notch signaling [11].

A disintegrin and metalloproteinase (ADAM) family is a group of multidomain transmembrane proteins with the activity of proteolytic enzymes that are involved in the regulation of many processes including: cell adhesion and migration, intercellular signaling, regulation of bioavailability of growth factors and cytokines, and proteolysis of the intercellular matrix. These processes, in the state of physiology, maintain the homeostasis of the human body, but also play an important role in the pathomechanism of neoplasm and the progression of neoplastic diseases [12].

Many previous studies have proven that ADAM10 and ADAM17 also play an important role in the development and progression of metabolic syndrome, and thus the development of inflammation, obesity and many diseases, including diabetes mellitus type 2 (DMT2) [10,13].

In addition, ADAM17 appears to be involved in several inflammatory diseases related to the cardiovascular system, such as atherosclerosis, ischemia and heart failure [14]. When considering CVD based on atherosclerosis, various key mediators have already been identified as substrates for ADAM10 and/or ADAM17 [15]. Among others, as such substrates, molecules of the vascular endothelial (VE)-cadherin and adhesion molecules A, which play a key role in vascular permeability and leukocyte transmigration, have been identified [16,17].

The article below is an original paper presenting the results of the determination of ADAM10 and 17 protein concentrations in CRC tissue and in the surgical margin and their correlation with selected clinical parameters including BMI, DMT2 and CVD.

## 2. Material and Methods

### 2.1. Study Population and Data Acquisition

The study group included 72 patients (36 men, 36 women) with CRC, hospitalized in the Department of General and Gastroenterological Surgery with the Oncological Surgery Subdivision, Bytom, Poland. During hospitalization, all patients underwent elective colorectal resections—while tumor specimens and macroscopically unchanged tissue fragments with dimensions of approximately 1 cm^3^ were collected. The samples were transported to the laboratory on ice, then the tissue pieces were washed with cold PBS buffer and weighed to prepare 10% tissue homogenates.

Anthropometric measurements were carried out to calculate the body mass index (BMI). In addition, comorbidities, age or sex were analyzed. Patients after surgical treatment and obtaining histopathological results were divided into 4 groups according to TNM Classification of Malignant Tumours, 8th based on clinical data, results of imaging studies and histopathological reports. Due to the small size of the study group, the division into 4 main grades was applied.

The characteristics of the study group are described in Table 1. The study was approved by the Bioethics Committee of the Medical University of Silesia (number PCN/0022/KB1/42/VI/14/16/18/29/20).

### 2.2. Preparation of Tissue Homogenates

To obtain 10% homogenates, a tissue fragment (30–60 mg) was ground in a PRO 200 mechanical homogenizer (PRO Scientific Inc., Oxford, CT, USA) at a speed of 10,000 RPM (5 times 1 min at 2-min intervals) in the presence of an appropriate volume of cooled buffer PBS (PBS without Ca and Mg; pH 7.4; BIOMED, Lublin, Poland) containing 0.5% Triton^®^ X-100 (Sigma-Aldrich^®^, St. Louis, MO, USA). The obtained homogenates were then centrifuged at 4000 RPM for 15 min at +4 °C. Supernatants were divided into appropriate aliquots and frozen at −80 °C until the further determination of ADAM10 and ADAM17 protein concentrations.

### 2.3. Total Protein Concentration Determinations

The AccuOrange™ Protein Quantitation Kit (Biotium, Fremont, CA, USA) was used to quantify total protein. Assays were performed in the tissue lysates according to the protocol provided with the kit, preparing 26-fold dilutions of the samples for protein content measurement. The detection range was 0.1–15 µg/mL protein. Fluorescence was measured at an excitation wavelength of 480 nm and an emission wavelength of 598 nm (SYNERGY H1 microplate reader; BIOTEK, Winooski, VT, USA using the GEN5 program). The determinations were made in triplicate. Total protein determinations were performed due to the necessity to express the concentration of ADAM10 and ADAM17 in units per µg of protein.

### 2.4. Determination of ADAM10 and ADAM17 Protein Concentrations

ADAM10 and ADAM17 protein concentrations in tissue homogenates were determined by enzyme immunoassay using ADAM10 and 17 assay kits (Cloud-Clone Corp., Houston, TX, USA) according to the manufacturer’s instructions. To determine the concentrations of the tested samples, a calibration curve was prepared using the standards included in the kit. Plates were read by Bio-Tek µQuant Universal Microplate Spectrophotometer (Bio-Tek, Winooski, VT, USA), using 450 nm as the primary wavelength. Data Analysis Software KCJunior (Bio-Tek, Winooski, VT, USA) was used. All standards and samples were run in duplicate. The absorbance was transformed into concentration.

The ADAM10 and 17 protein concentrations for each sample were normalized to the total amount of protein in the tissue lysates. Values are expressed in pg/µg protein for ADAM10 and for ADAM17 in ng/µg protein.

All immunoassays were performed at the Department of Medical and Molecular Biology, Faculty of Medical Sciences in Zabrze, Medical University of Silesia in Katowice.

### 2.5. Statistical Analysis

Analysis was performed using Statistica version 13.3.

The patients were divided into groups according to the stage of cancer, tumor location, BMI value and coexistence of diseases (DMT2, CVD-hypertension, atherosclerosis and ischemic heart disease).

The results are reported as the mean and standard error of the mean (SEM). Shapiro–Wilk’s W test was used to assess the normality of the distribution. Parametric tests were used for normally distributed variables, otherwise nonparametric tests. To compare the concentrations of ADAM10 and ADAM17 between the groups, Student’s *t*-test for normally distributed variables and the Mann–Whitney U test for variables deviating from the normal distribution were used. Differences at *p* < 0.05 were considered statistically significant.

## 3. Results

The study population included 36 men and 36 women. There were no significant differences in age and BMI between the two groups. The concentration of ADAM proteins was analyzed in 4 major clinical and histopathological stages. Additionally, the coexistence of DMT2 and CVD was taken into account. No patients were diagnosed with IBD.

### 3.1. ADAM10 Protein Concentration

The performed analyses showed a lower protein concentration of ADAM10 in the surgical margin in the IV stage of clinical and histopathological advancement than in stages I-III (219.04 vs. 262.94 pg/µg protein; *p* < 0.05). In the first degree, a higher protein concentration of ADAM10 was demonstrated in the tissue of the surgical margin than in the tumor (282.78 vs. 203.5; *p* < 0.05).

The mean protein concentration of ADAM10 for the entire study group was higher in the surgical margin tissue than in the tumor tissue (249.34 vs. 228.82 pg/µg protein), but the results were not statistically significant (*p* = 0.22). The mean protein concentration of the ADAM10 was higher in men, both in the tumor and in the margin—these differences, however, were not statistically significant. There were also no statistically significant differences between the protein concentration of ADAM10 (both in the tumor and in the surgical margin) in obese and non-obese patients as well as in DMT2 and patients with CVD compared to patients without these diseases. In the tumor, a higher protein concentration of ADAM10 was detected in this group of patients, but without statistical significance (244.64 vs. 222.18 pg/µg protein; *p* = 0.45).

### 3.2. ADAM17 Protein Concentration

**Figure 1:**
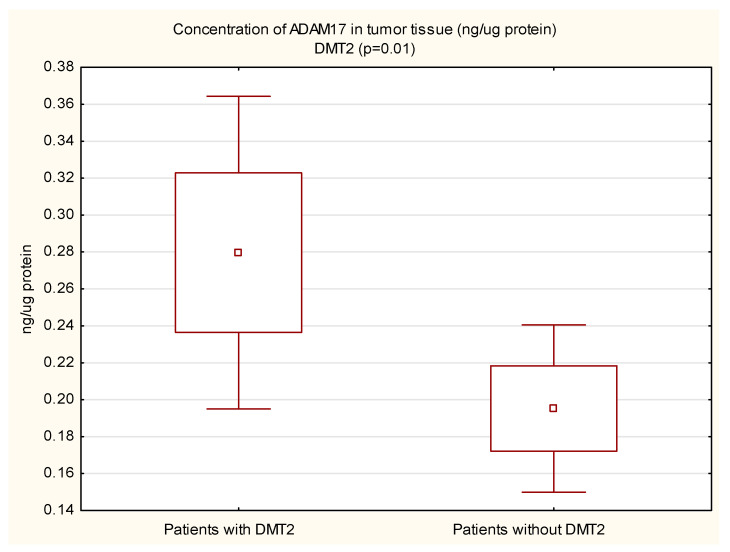
Concentration of ADAM17 protein in tumor tissue in CRC patients with and without coexisting diabetes mellitus type 2 (DMT2).

**Figure 2:**
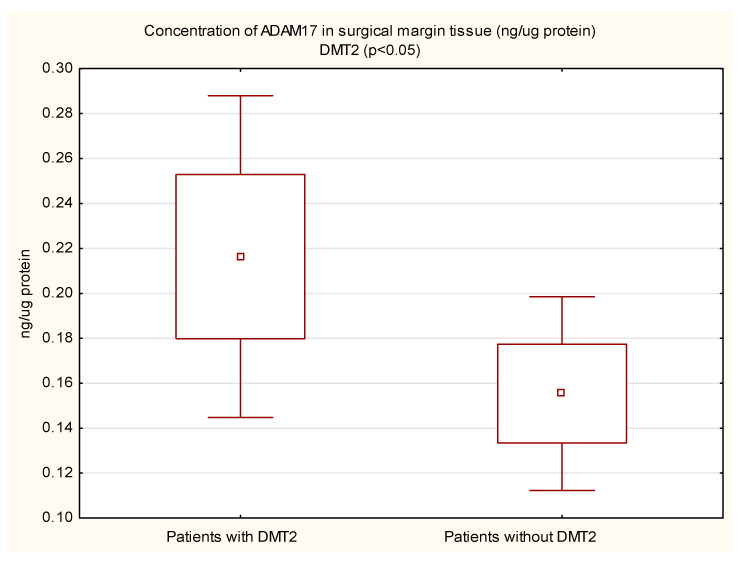
Concentration of ADAM17 protein in margin tissue in CRC patients with and without coexisting DMT2.

**Figure 3:**
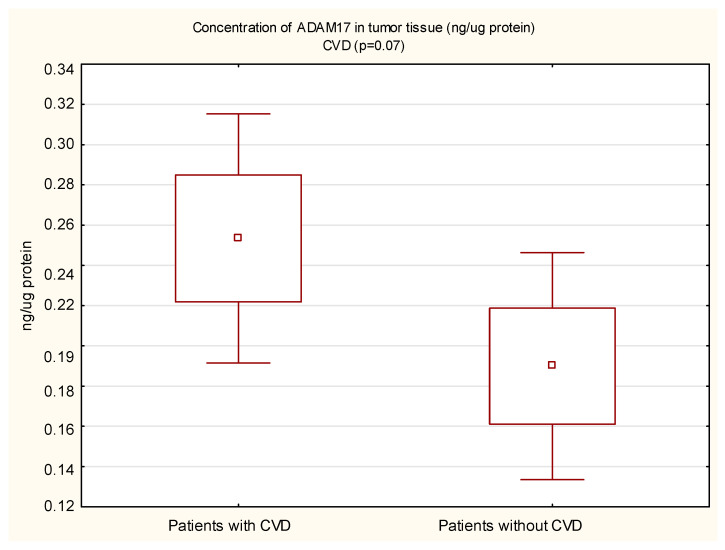
Concentration of ADAM17 protein in tumor tissue in CRC patients with and without cardiovascular diseases (CVD).

**Figure 4:**
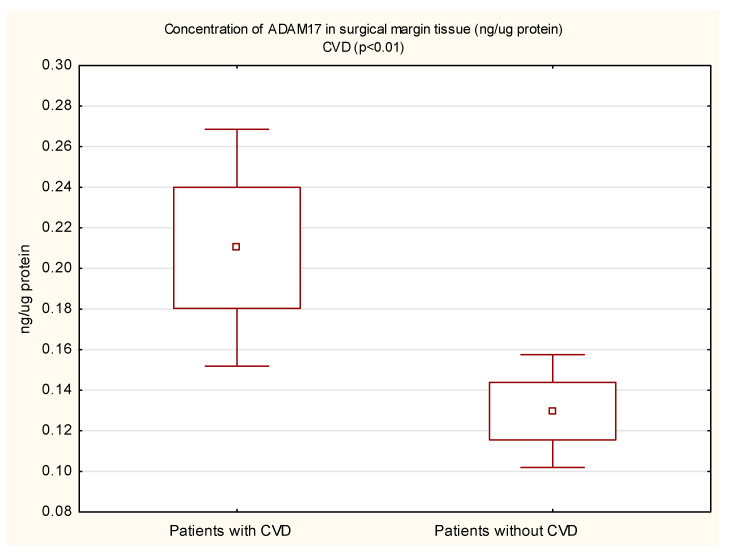
Concentration of ADAM17 protein in tumor tissue in CRC patients with and without coexisting CVD.

The mean protein concentration of ADAM17 in the margin tissue was lower in women than in men, and the result was statistically significant (0.14 vs. 0.22; *p* < 0.05). In the tumor, the protein concentration of ADAM17 was also lower in women, but not statistically significant. The protein concentration of ADAM17 in tumor tissue (0.28 vs. 0.20 ng/µg protein, *p* = 0.01) and in the surgical margin (0.22 vs. 0.16 ng/µg protein, *p* < 0.05) of diabetic patients was higher than in non-diabetic patients. These results are shown in Figure 1 and Figure 2. In patients with concomitant CVD, a statistically significantly higher protein concentration of ADAM17 in the margin tissue was demonstrated (0.21 vs. 0.13 ng/µg protein, *p* < 0.01); in the tumor, the protein concentration was also higher, but the necessary statistical significance point was not reached (0.25 vs. 0.19 ng/µg protein, *p* = 0.05). These results are shown in Figure 3 and Figure 4.

The mean protein concentration of ADAM17 for the study group was higher in the tumor than in the margin tissue (0,23 vs. 0.18 ng/µg protein, *p* = 0.09), but the results were not statistically significant. ADAM17 protein concentration in both tumor and margin tissue did not differ in obese and non-obese subjects (statistically insignificant differences)—but in tumors, the mean ADAM17 protein concentration in obese patients was higher than in subjects without obesity. There were no statistically significant differences between ADAM17 protein concentration in stage IV compared to earlier stages (I–III), both in tumors and in the surgical margins.

The results of the analyses are presented in Table 2.

## 4. Discussion

Both ADAM10 and ADAM17 have already been reported to be potentially involved in the development of gastrointestinal cancers. One available study showed high levels of ADAM10 and ADAM17 transcripts in gastric adenocarcinoma [18]. Another report indicates a relationship between ADAM10 in gastric cancer and worsened prognosis through a positive correlation with tumor size, infiltration depth, vascular invasion, lymph node involvement or the presence of distant metastases [19]. A similar relationship was demonstrated for ADAM17 [20,21]. Moreover, high levels of ADAM17 expression can cause gastric cancer progression through the Notch and/or Wnt signaling pathway [22] and also through the epidermal-like growth factor (EGF) pathway [23]. Studies have shown also the overexpression of ADAM17 in the tumor tissue of esophageal cancer [24], as well as the participation of this protein in the pathogenesis of pancreatic cancer [25].

ADAM10 and ADAM17 are important, well-known links in the development and progression of CRC. Particularly noteworthy are studies of the correlation between the occurrence of CRC with metabolic disorders and the presence of chronic inflammation. ADAM10 modulates the inflammatory response pathways by influencing the Notch signaling pathway [26]. A correlation between ADAM10 expression and the stage of CRC has also been demonstrated [27]. ADAM 17 is involved in the stimulation and maintenance of the inflammatory process by the production of cytokines, chemokines, endothelial adhesion molecules, and the increase in vascular permeability, releasing the active tumor necrosis factor TNF-α [8,28]. In addition, ADAM17 causes tumor growth by activating growth factors from the EGF family [29], as well as by influencing angiogenesis and secretion of cytokines such as IL-6, IL-10, IL-12, or the above-mentioned TNF-α [30].

The most important result of our work is that the protein concentration of ADAM17 in the tissue of both the CRC tumor and the surgical margin is higher in CRC patients with comorbidities such as DMT2 or CVD in comparison to CRC patients without these diseases. This is confirmed by previous studies on the role of ADAM17 in the development of metabolic syndrome, inflammation and CVD [10,13,14]. It should be emphasized that ADAM17 has a documented role in the formation of the inflammatory process and, consequently, in diseases that are caused by chronic inflammation, such as DMT2, ischemic heart disease or CRC. Chronic inflammation leads to the development of CRC by over-activating immune cells that secrete cytokines such as TNF, IL-17, IL-23 and IL-6, which lead to the propagation of the inflammatory environment and possibly the development of precancerous conditions [28,31]. Among other things, in this mechanism, the presence of IBD can lead to the development of CRC. The genomic background of the colitis-associated carcinoma also appears to have a mutation associated with Wnt pathway signaling [32]. The hypothesis about the relationship between inflammation and neoplasm is also confirmed by a study on animal models, in which the use of substances with anti-inflammatory properties inhibited the proliferation of CRC [33].

Moreover, the protein concentration of ADAM17 in the whole study group was higher in CRC tissue than in the tissue of the surgical margin, although the result did not reach statistical significance, it could suggest an important role in the pathogenesis of CRC. Many previous studies confirm this observation. As early as 2005, scientists showed ADAM17 overexpression in primary human CRC [29]. Walkiewicz et al. showed that the level of ADAM10 and ADAM17 in the blood serum is higher in patients with CRC [34]. Other studies on the role of ADAM17 in CRC have shown that ADAM17 cellular levels can increase the mobility of cancer cells and the expression of proangiogenic factors that can determine tumor progression and metastasis [35]. Effective attempts to inhibit the activity of ADAM17 by the use of specific antibodies confirm the important role of this protein in patients with CRC [36,37].

An interesting result of our analysis is the higher protein concentration of ADAM10 in the tissue of the surgical margin than in the tissue of CRC. In our opinion, ADAM10 could play an important role in the very early stages of cancer. This hypothesis may be confirmed by our subsequent results—a higher protein concentration of ADAM10 in the tissue of the surgical margin than in the tumor tissue in patients with stage I of CRC, and a generally higher protein concentration of ADAM10 in the tissue of the surgical margin in patients with stages of cancer earlier than IV. We also know that ADAM10 is involved in the pathogenesis of CRC by influencing the Notch signaling pathway, which under physiological conditions helps, among other things, to control intestinal damage, but in the case of dysregulation, it can lead to the formation and progression of CRC [26].

In conclusion, the most important deduction of our work is that the protein concentration of ADAM17 was significantly higher in both tumor and margin in patients with CRC with coexisting DMT2 in comparison to CRC patients without DMT2. In addition, our results showed that ADAM17 protein concentration was significantly higher in the surgical margin in patients with coexistence of CVD in relation to patients without these comorbidities. The analysis of our results also demonstrated that the concentration of ADAM17 protein in the study group was higher in the CRC tissue than in the tissue of the surgical margin and the concentration of ADAM10 protein in the surgical margin tissue was higher than in the CRC tissue.

The study presents preliminary results of our research. The work has some limitations which include the small size of the studied group. Moreover, it seems that many factors other than the presence of CRC or the comorbidities that we analyzed may have an influence on the concentration of ADAM10 and 17 in tissues. However, the obtained results allow us to hope for their practical application in the diagnosis, treatment and follow-up of CRC. Future investigations are necessary to validate the kinds of conclusions that can be drawn from this study.

## Figures and Tables

**Table 1:** The characteristics of the study group.

Characteristics	All Patients	Male	Female
*n*	72	36	36
Age (years)	66.29 ± 10.03	64.47 ± 9.83	68.11 ± 10.04
BMI (kg/m^2^)	27.44 ± 4.42	28.36 ± 3.79	26.53 ± 4.85
I stage (*n*)	13	6	7
II stage (*n*)	16	9	7
III stage (*n*)	21	7	14
IV stage (*n*)	22	14	8
DMT2 (*n*)	29	16	13
CVD (*n*)	44	24	20

*n*—number; BMI—body mass index; DMT2—diabetes mellitus type 2; CVD—cardiovascular diseases.

**Table 2:** ADAM10 and ADAM17 proteins concentration in the study group according to analyzed data.

		ADAM10 (pg/µg Protein)		ADAM17 (ng/µg Protein)	
		Tumor	Surgical Margin	Tumor	Surgical Margin
Study group (*n* = 72)		228.82 ± 112.33	249.34 ± 83.44	0.23 ± 0.19	0.18 ± 0.16
Stage of colorectal cancer	CSI (*n* = 13)	203.5 ± 118.09	282.78 ± 69.42	0.22 ± 0.19	0.12 ± 0.05
	CSII (*n* = 16)	206.48 ± 113.73	266.63 ± 78.65	0.23 ± 0.17	0.22 ± 0.21
	CSIII (*n* = 21)	245.7 ± 120.84	248.03 ± 104.37	0.25 ± 0.22	0.18 ± 0.14
	CSIV (*n* = 22)	244.64 ± 100.57	219.04 ± 63.92	0.23 ± 0.16	0.18 ± 0.2
Sex	Female (*n* = 36)	215.40 ± 109.86	237.93 ± 82.97	0.21 ± 0.15	**0.14 ± 0.13 ^a^**
	Male (*n* = 36)	241.88 ± 114.7	260.43 ± 83.55	0.25 ± 0.22	**0.22 ± 0.19 ^a^**
BMI	≥30 kg/m^2^ (*n* = 21)	229.79 ± 96.58	253.49 ± 96.57	0.26 ± 0.23	0.18 ± 0.14
	<30 kg/m^2^ (*n* = 51)	228.44 ± 118.83	247.71 ± 78.7	0.22 ± 0.17	0.18 ± 0.17
DMT2	Yes (*n* = 29)	241.78 ± 124.54	250.12 ± 77.68	**0.28 ± 0.23 ^b^**	**0.22 ± 0.2 ^a^**
	No (*n* = 43)	226.83 ± 101.75	254.43 ± 86.49	**0.2 ± 0.15 ^b^**	**0.16 ± 0.14 ^a^**
CVD	Yes (*n* = 44)	243.76 ± 115.59	258.19 ± 77.91	0.25 ± 0.21	**0.21 ± 0.19 ^c^**
	No (*n* = 28)	214.93 ± 102.37	242.82 ± 77.91	0.19 ± 0.15	**0.13 ± 0.07 ^c^**

^a^*p* < 0.05; ^b^ *p* = 0.01; ^c^ *p* < 0.01. The other analyzed results were not statistically significant (*p* > 0.05).

## Data Availability

The data presented in this study are available on request from the corresponding author. The data are not publicly available due to the protection of patient data.

## Abbreviations

CVD	Cardiovascular diseases
CRC	Colorectal cancer
DMT2	Diabetes mellitus type 2
IBD	Inflammatory bowel diseases
CAC	Colitis-associated cancer
IGF	Insulin-like growth factor
TNFα	Tumor necrosis factor α
VEGF	Vascular endothelial growth factor
EGF	Epidermal growth factor
FGF	Fibroblast growth factor
TGFα	Transforming growth factor-alpha
IL-6	Interleukin 6
ADAM	A disintegrin and metalloproteinase
ELISA	Enzyme-linked immunosorbent assay
BMI	Body mass index
